# Interaction of the chemotherapeutic agent oxaliplatin and the tyrosine kinase inhibitor dasatinib with the organic cation transporter 2

**DOI:** 10.1007/s00204-024-03742-1

**Published:** 2024-04-08

**Authors:** Sara Ahmed Eltayeb, Julia M. Dressler, Lukas Schlatt, Moritz Pernecker, Ute Neugebauer, Uwe Karst, Giuliano Ciarimboli

**Affiliations:** 1 Medizinische Klinik D, Experimentelle Nephrologie, Universitätsklinikum Münster, Albert-Schweitzer-Campus 1/A14, 48149 Münster, Germany; 2 Institut Für Anorganische Und Analytische Chemie, Universität Münster, Münster, Germany

**Keywords:** Transporters, Oxaliplatin, Peripheral neurotoxicity, Dasatinib

## Abstract

Oxaliplatin (OHP) is effective in colorectal cancer treatment but induces peripheral neurotoxicity (OHP-induced peripheral neurotoxicity, OIPN), diminishing survivor quality of life. Organic cation transporter 2 (OCT2) is a key OHP uptake pathway in dorsal root ganglia. Competing for OCT2-mediated OHP uptake, such as with the tyrosine kinase inhibitor dasatinib, may mitigate OHP side effects. We investigated OHP and dasatinib interaction with OCT2 in human embryonic kidney 293 (HEK293) cells expressing OCT2 within a 10^–3^ to 10^–7^ M concentration range. Uptake competition experiments using fluorescent organic cation 4-(4-dimethylaminostyryl)-N-methylpyridinium (ASP^+^, 1 µM) and mass spectrometry (MS) to determine cellular platinum content indicated that OHP (100 µM) is an OCT2 substrate, mediating OHP cellular toxicity. ASP^+^ and MS analysis revealed dasatinib as a non-transported inhibitor of hOCT2 (IC_50_ = 5.9 µM) and as a regulator of OCT2 activity. Dasatinib reduced transporter V_max_, potentially via Y544 phosphorylation suppression. MS analysis showed cellular dasatinib accumulation independent of hOCT2. Although 3 µM dasatinib reduced 100 µM OHP accumulation in hOCT2-HEK293 cells, co-incubation with dasatinib and OHP did not prevent OHP toxicity, possibly due to dasatinib-induced cell viability reduction. In summary, this study demonstrates OHP as an OCT2 substrate and dasatinib as a non-transported inhibitor and regulator of OCT2, offering potential for OIPN mitigation.

**Supplementary Information:**

The online version contains supplementary material available at 10.1007/s00204-024-03742-1.

## Introduction

**Fig. 1:**
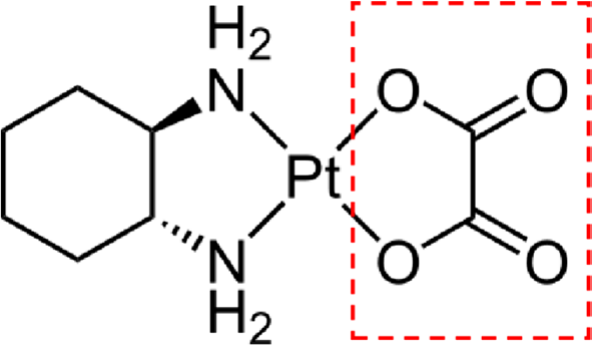
Chemical structure of oxaliplatin. The dashed box indicates the oxalate leaving groups

After cisplatin (first generation) and carboplatin (second generation), oxaliplatin (OHP) is a third-generation platinum-based chemotherapy drug used to treat various types of cancer, particularly colorectal cancer. The OHP (oxalato(trans-l-1,2-cyclohexanediamine)platinum(II)) molecule is characterised by the presence of an oxalate 'leaving group' and the diaminocyclohexane carrier ligand (see Fig. 1) (Alcindor and Beauger 2011). In blood plasma, the oxalate leaving groups of OHP are replaced by Cl^−^ and on entering the cell, where the concentration of chloride is much lower than in blood plasma (10 vs 100 mM), the Cl^−^ are replaced by water molecules to give the pharmacologically active form (Shoeib and Sharp 2012). In cells, these H_2_O molecules are relatively easily displaced and OHP can bind to proteins and DNA, forming various types of cross-links with DNA (intra- and interstrand adducts) and proteins (Corno and Perego 2018). A special feature of OHP-DNA-adducts is that, because of their conformation, they are not recognised by the DNA mismatch repair (MMR) machinery (Corno and Perego 2018). The formation of OHP-DNA- and -protein-crosslinks is the basis for the antiproliferative, apoptotic, and toxic effects of OHP (Alcindor and Beauger 2011).

OHP is indicated for the adjuvant treatment of stage III colorectal cancer following resection of the primary tumour and for the treatment of metastatic colorectal cancer (Devanabanda and Kasi 2023). OHP has been approved by the U.S. Food and Drug Administration for use in combination with infusional 5-fluorouracil and leucovorin in a regimen known as FOLFOX (Meyerhardt and Mayer 2005). In paediatric patients, OHP is used in combination with several other chemotherapeutic agents to treat refractory or relapsed neuroblastoma and solid tumours (Hartmann et al. 2011; Lam et al. 2015; Tran et al. 2015).

As with most chemotherapeutic agents, OHP can cause a number of side effects, some of which can be severe. The most common adverse effects of chemotherapy with OHP are acute and chronic peripheral neuropathy, characterised by paresthesia, hypoesthesia and dysesthesia, exacerbated by cold (Cersosimo 2005). Acute and chronic peripheral neuropathy develop in 90% and 50%, respectively, of patients treated with OHP (Cersosimo 2005). Interestingly, in opposition to chemotherapy with cisplatin, OHP treatment does not induce significant nephrotoxicity. Acute OHP-induced peripheral neurotoxicity (OIPN) resolves rapidly and appears to be associated with changes in the expression and function of ion channels, such as Ca^2+^, Na^+^, and K^+^ channels, transient receptor potential ankyrin 1 (TRPA1) and TRP melastatin 8 (TRPM8) [these mechanisms are briefly summarized in (Fujita et al. 2019)]. Chronic OIPN is thought to be caused by OHP accumulation in dorsal root ganglia (DRG), leading to neuronal injury (Ta et al. 2006). The transport of OHP across the plasma membrane of DRG cells has been shown to be mediated by transporters, especially by those that are physiologically involved in the transmembrane movement of organic cations. This class of transport proteins includes organic cation transporters (OCT), organic cation transporter novel (OCTN) and multidrug and toxin extrusion protein (MATE). Interestingly, these transporters are described as polyspecific, because they can accommodate substrates with very different chemical structures in their binding region. This polyspecificity is due to the particular structure of the binding pocket of OCT, which is thought to be very large and to contain specific, partially overlapping interaction domains for different substrates (Volk et al. 2003; Koepsell et al. 2007; Koepsell 2019; Keller et al. 2019). Human OCT1 (hOCT1) and hOCT2 have been shown to transport OHP into cells in vitro (Zhang et al. 2006). OCT2 has been shown to be expressed in mouse and human DRG and to be important for OINP development in a mouse model (Sprowl et al. 2013; Huang et al. 2020). OCTN1/2 and MATE1 are also able to mediate OHP cellular accumulation (Fujita et al. 2019). In mice, OIPN has been found to be associated also with OCTN1-mediated uptake of OHP into the DRG, whereas MATE1-mediated efflux of OHP from the DRG appears to have a protective function (Fujita et al. 2019), analogous to that observed in the kidneys (Yokoo et al. 2007). Even in the rat, OCTN1/2 are functionally present in DRG neurons, where OCTN1 mediates OHP accumulation (Jong et al. 2011). Copper transporters such as Ctr1 and ATP7a have also been implicated in OHP transport in DRG and OIPN (Liu et al. 2009; Ip et al. 2010).

According to a recent systemic review and meta-analysis of the literature (Peng et al. 2022), there is still no clinically efficient therapeutic protocol able to avoid or reduce OIPN. This may also be due to the difficulty of selectively protecting non-target cells, rather than cancer cells, from OHP cytotoxicity. Since membrane transporters have a specific tissue distribution, with some of them being well expressed only in differentiated and not in undifferentiated cells, they are potentially excellent targets for protection against OIPN: competition with OHP transport in the DRG may reduce OHP accumulation and OIPN development in these cells. Indeed, there are already indications that competing OHP uptake by OCT in vitro by the antiacid cimetidine (Zhang et al. 2006) or in vivo by the tyrosine kinase inhibitor dasatinib (Huang et al. 2020) may decrease cellular OHP-accumulation and toxicity by OCT.

In this study, we investigated the possibility of reducing the cellular toxicity of OHP in vitro. In addition, the interaction mechanisms of OHP and dasatinib with OCT2 were characterised.

## Materials and methods

### Cell culture

The experiments utilized human embryonic kidney (HEK)293 cells (CRL-1573; American Type Culture Collection, Rockville, MD), which were in the wild-type form (WT) or were engineered to stably express mouse (m) or human (h) OCT1-2 or hMATE1. The cells were cultivated at 37 °C in 50 mL cell culture flasks (Greiner, Frickenhausen, Germany) using DMEM (Biochrom, Berlin, Germany) supplemented with 3.7 g/L NaHCO_3_, 1.0 g/L D-glucose, and 2.0 mM l-glutamine (Biochrom), and incubated with 5% CO_2_. The growth medium included 100 U/mL penicillin, 100 mg/L streptomycin (Biochrom), 10% fetal calf serum, and, for hOCT1-2 and mOCT1-2 transfected cells, 0.8 mg/mL geneticin (PAA Laboratories, Coelbe, Germany), for hMATE1-transfected cells, hygromycin B (0.5 mg/mL; Roth, Karlsruhe, Germany). Establishment of HEK293 cells transfected with hOCT1-2, mOCT1-2, and hMATE1 has been described in Lee et al. (2009), Schmidt-Lauber et al. (2012), and Schlatter et al. (2014), respectively. Experiments were conducted with confluent cells grown in 96-well microplates for 2–3 days, sourced from passages 12–60. No significant variation in the time required to achieve confluency was observed within this wide range of passages. The culture and functional analyses of these cells were granted approval by the state government Landesumweltamt Nordrhein-Westfalen, Essen, Germany (no. 521.–M-1.14/00).

### 4-(4-Dimethylaminostyryl)-N-methylpyridinium (ASP^+^) uptake

To investigate the potential interaction of oxaliplatin (OHP) and dasatinib with transporters for organic cations, a dynamic cis-inhibition protocol of ASP^+^ uptake was used. ASP^+^ is a known fluorescent substrate for all the examined transporters. The experiments were carried out with HEK293 cells seeded in 96-well plates and allowed to grow to 80–100% confluence. Dasatinib (Biozol, Eching, Germany) was dissolved in dimethyl sulfoxide (DMSO). Dasatinib and OHP (Teva Pharm, Ulm, Germany) solutions in the concentration range of 10^−3^ to 10^−7^ M were prepared with ringer-like solution (RLS) that lacked bicarbonate (HCO_3_^−^ free Ringer-like solution). The RLS contained the following components (in mmol/l): NaCl 145, K_2_HPO_4_ 1.6, KH_2_PO_4_ 0.4, D-glucose 5, MgCl_2_ 1, and calcium gluconate 1.3, with a pH adjusted to 7.4. Fluorescence measurements were performed using the TECAN Infinite F200 (Tecan, Maennedorf, Switzerland) microplate reader. The transporter function was investigated by measuring the slope of fluorescence emission, detected at 590 nm after excitation at 450 nm, in the first 60 s after the addition of ASP^+^ at a final concentration of 1 µM (10 µM when using hMATE1-expressing cells). The ASP^+^ uptake without any potential inhibitor was set at 100%, and fluorescence was measured in each well before and after the addition of ASP^+^. To specifically study the regulation of hOCT2, ASP^+^ uptake was evaluated after 10 min incubation with dasatinib and compared to control experiments conducted under the same conditions without dasatinib. The results are expressed as the percentage change of ASP^+^ uptake relative to the control experiments. All the experiments were conducted at a temperature of 37 °C to mimic physiological conditions and ensure the relevance of the findings.

### Mutagenesis of hOCT2

The full-length hOCT2 (SLC22A2, NM 003058.3) cloned in the expression vector pEGFP-N3 (Clontech-Takara Bio Europe, Saint-Germain-en-Laye, France), as previously described (Brast et al. 2012), was used to obtain the hOCT2 mutants Y544A. The mutation was accomplished by using the quick-change site-directed mutagenesis kit (Stratagene, La Jolla, CA, USA) following the instructions of the manufacturer and using the primers listed in Supplementary Materials Table 1. The successful mutation was verified by automated sequencing. The Y544A-hOCT2 was transfected into HEK293 cells using the Turbofect Transfection Reagent following the protocol of the manufacturer (Thermo Scientific, Rockford, IL, USA).

**Table 1:** Limit of detection (LOD) and limit of quantification (LOQ) of dasatinib quantification via LC–MS and oxaliplatin quantification via ICP-MS. Values are referred to the pure cell lysate

	Dasatinib	Oxaliplatin
LOD/ng mL^−1^	1	30
LOQ/ng mL^−1^	3	100

### Measurement of cytotoxicity

The cytotoxicity was assessed using a modified 3-(4,5-dimethylthiazol-2-yl)-2,5-diphenyltetrazolium bromide (MTT) assay, which examined the response of HEK293-WT, -hOCT2, and -hMATE1 cells to OHP treatment. The assay determined the glycolysis rate by measuring the reduction of the yellow tetrazolium salt MTT to a purple formazan dye. To conduct the experiment, the cells were grown for 24 h before adding OHP-containing cell culture medium to achieve a concentration of 100 µM OHP (this OHP concentration was chosen because it caused a significant decrease in cell viability, see Supplementary Material Fig. 1). Moreover, OHP C_max_ and AUC in patients are ≈ 10 µM and 140 µM/h, respectively (Burz et al. 2009)). The control cells were treated with OHP-free complete cell culture medium. The cells were incubated with OHP for 10 min, after which the medium was replaced with fresh complete cell culture medium. After 48 h incubation, 5 mg/ml MTT solution per well was added, and the cells were incubated for additional 3 h. Next, the medium was gently removed, and 100 μl of lysis buffer, containing 10% (w/v) sodium dodecyl sulfate and 40% (v/v) dimethylformamide per well, was added to dissolve the blue formazan dye after shaking the plates for 10 min to disrupt the cell structure. Finally, the absorbance was measured at 590 nm using an automated microtiter plate reader (Infinite M200; Tecan, Männedorf, Switzerland) to determine the percentage of viable cells in the untreated controls compared to those treated with OHP. In some experiments, dasatinib was added at a final concentration of 3 µM alone or along with OHP to investigate its potential protective effect against OHP-induced cytotoxicity. In some experiments with hMATE1-HEK293 cells, acidification with NH_4_Cl as described below was performed before incubation with OHP.

### Determination of platinum cellular accumulation by inductive coupled mass spectrometry (ICP-MS)

Platinum accumulation was assessed following the procedure outlined in reference (Wehe et al. 2014) in collaboration with the Institute of Inorganic and Analytical Chemistry at Münster University. For this study, HEK293 cells with stable transfections of empty vector (called wild type cells, WT), hOCT2, or hMATE1 were seeded on a 24-well plate. Once the cells reached 80 to 90% confluency, the culture medium was replaced with RLS at pH 7.4, and the cells were exposed to 100 μM of OHP for 10 min at 37 °C. After the incubation period, the medium was removed, and the cells were rapidly rinsed with ice-cold RLS. Subsequently, cell solubilization was achieved using either 0.1% formic acid or hypoosmotic lysis with water. The resulting solution was then subjected to centrifugation, and the Platinum content in the supernatant was determined using ICP-MS at the Institute of Inorganic Chemistry at Münster University. Platinum concentration was calculated based on an external calibration curve, as described below, and the obtained values were normalized according to the protein content of the cells measured using a Bradford assay (Bradford 1976). To further underscore the assertion that OHP is a substrate of hOCT2, saturation experiments were undertaken. Cellular accumulation of OHP was assessed following incubation with OHP concentrations ranging from 50 to 900 µM. The evaluation of OHP cellular accumulation was conducted both in the absence (total OHP uptake) and presence (unspecific OHP uptake) of 1 mM TPA^+^, a potent inhibitor of hOCT2 transport. By subtracting the unspecific from the total OHP uptake, the specific (hOCT2-mediated) OHP uptake could be precisely calculated. *Calibration and sample preparation*: Matrix-matched standards were prepared using untreated HEK293 cell lysate and platinum ICP-MS standard solution (Inorganic Ventures^©^, Christiansburg, VA, USA). Iridium ICP-MS-solution (Fluka Chemie GmbH, Buchs, Switzerland) served as internal standard (IS). 50 µl IS solution and 50 µl external standard solutions were added to 25 µl lysate. The mixture was digested in 500 µl HNO_3_ (VWR™ International S.A.S., Rosny-sous-Bois, France) (50% v/v) for 1 h at 100 °C. The digests were diluted to 5 ml with MilliQ water (18.2 kΩ) (Merck KgaA, Darmstadt, Germany) subsequently, resulting in an end concentration of 0, 0.1, 0.5, 1, 5, 10, and 25 ng ml^−1^ for the external calibration standards and 1 ng/ml IS. All cell samples were equally prepared to the calibration standards. *Instrumental set up:* quantification of OHP was done by total Platinum determination using an Agilent 7700 ICP-MS system (Agilent Technologies, Santa Clara, CA, USA). Samples were injected directly into the ICP-MS system with a pump rate of 0.1 revolutions per second (rps) and a nebulizer gas flow of 1.05 l min^−1^. Radio frequency (RF) power was 1550 W. Platinum was measured at *m*/*z* 195 and iridium at *m*/*z* 193. Each sample and external standard was measured in triplicates.

### Quantification of dasatinib by liquid chromatography mass spectrometry (LC–MS)

*Calibration and sample preparation*: Matrix-matched standards were prepared using untreated HEK293 cell lysate. Dasatinib-d_8_ (Biomol GmBH^©^, Hamburg, Germany), served as IS. 10 µl of each, external standard solution of dasatinib (Merck^©^ KgaA, Darmstadt, Germany) and IS solution, were added to 25 µl cell lysate. After adding 50 µL acetonitrile (ACN) (VWR™ International S.A.S., Rosny-sous-Bois, France) the samples were centrifuged at 18,000 g and 4 °C for 35 min (BS Cryo, Serva Electrophoresis GmbH^©^, Heidelberg, Germany). 75 µl of the supernatant were removed and diluted 1:1 with MilliQ water. The final concentration of the IS was 10 ng ml^−1^ and the final concentrations of the external calibration standards were 0, 1, 5, 10, 50, and 100 ng ml^−1^. All cell samples were equally prepared to the calibration standards. *Instrumental set up*: Measurements were performed using an EVOQ® triple quadrupol mass spectrometer (Bruker Daltonics®, Bremen, Germany) system hyphenated to an Advanced Ultra-High Performance Liquid Chromatography (UHPLC) OLE (Bruker Daltonics®). The separation of the analyte from interferring cell components were performed on an Accurore C18 column (50 mm × 3 mm, 2.6 µm) (ThermoFischer Scientific™, Bremen, Germany). The mobile phase was H_2_O (A) and ACN (B) with 0.1% formic acid (Th. Geyer GmbH & Co. KG^©^, Renningen, Germany) respectively. Optimal peak shape and intensity were obtained with a flow rate of 1 ml min^−1^ and a gradient starting with 2% B for 15 s, then increasing B to 65% within 135 s, holding 65% for 15 s and finally equilibrate for 45 s at 2% B. The total run time was 210 s. The heated electrospray ionization (HESI) source was operated in positive mode, with a spray voltage of 4000 V and a probe temperature of 350 °C. The probe gas flow was set to 45 arbitrary units (AU) and the nebulizer gas flow was 55 AU. The detection of dasatinib and dasatinb-d_8_ was carried out in multiple-reaction-monitor (MRM) mode, with a transition of *m*/*z* 488 → *m*/*z* 401 for dasatinib and *m*/*z* 496 → *m*/*z* 406 for dasatinb-d_8_ at a collision energy of 30 eV respectively. Each sample and external standard was measured in triplicates. *Quality control and Quality assurance:* Relative standard deviations (RSDs) from technical triplicates ranged between < 1% and 8% in all samples and standards. Limit of Detection (LOD) and limit of quantification (LOQ) were determined via 3 sigma and 10 sigma criteria (Table 1).

### Cell acidification

As MATEs are pH-dependent transporters, their activity can be studied as a function of cellular pH. For this reason, hMATE1-HEK293 cells were acidified by incubation with NH_4_Cl as already described in (Kantauskaitė et al. 2020). Briefly, hMATE1-HEK293 cells were seeded on a 96-well plate and allowed to reach 90% confluence. The cells were then incubated with RLS in the presence or not of 30 mM NH_4_Cl at pH 7.4 and 37 °C for 20 or 30 min. The incubation solution was then removed and replaced with RLS. For ASP^+^ uptake experiments, 10 µM ASP^+^ was then added to RLS in the presence or absence of 1 mM cimetidine to test the specificity of the effect, and the uptake rate of ASP^+^ was measured. For toxicity testing, cells were incubated with 100 µM OHP for 5 min after removal of NH_4_Cl. Afterwards, OHP was removed, and cells were further incubated for 48 h before toxicity testing.

### Statistics

Data were analyzed with the help of using GraphPad Prism, Version 10.0 (GraphPad Software, Inc., San Diego, CA, USA). Data are plotted as individual data points with the mean ± SEM in the case they are normally distributed, or with the median of observations and the interquartile range in the case, they are not normally distributed. Unpaired two-sided Student’s *t* test and two-way ANOVA were used as appropriate to prove the statistical significance of the effects. Significance was inferred at the *p* < 0.05 level.

## Results

**Fig. 2:**
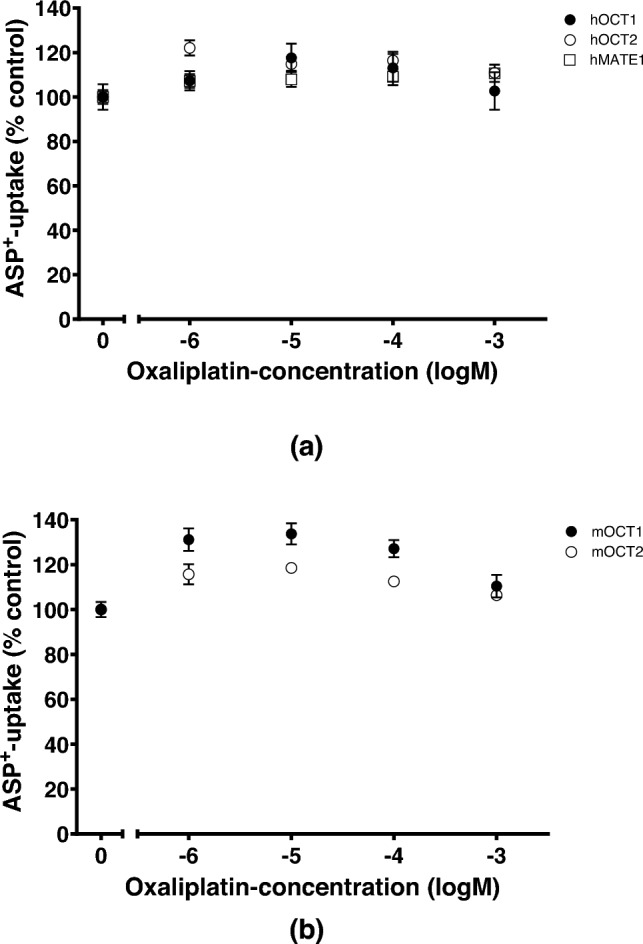
Inhibition of ASP^+^ uptake (1 µM) by increasing concentrations of OHP (10^–6^ to 10^–3^ M). **a** Result of experiments performed using HEK293 stably expressing hOCT1, hOCT2, or hMATE1. **b** Results of experiments performed in HEK293 cells stably expressing mOCT1 or mOCT2. A slight stimulation of ASP^+^ uptake was observed at low OHP concentrations. No inhibition of ASP^+^ uptake was observed. Each point represents the mean ± SEM of 6–24 replicates measured in at least 3 independent experiments

**Fig. 3:**
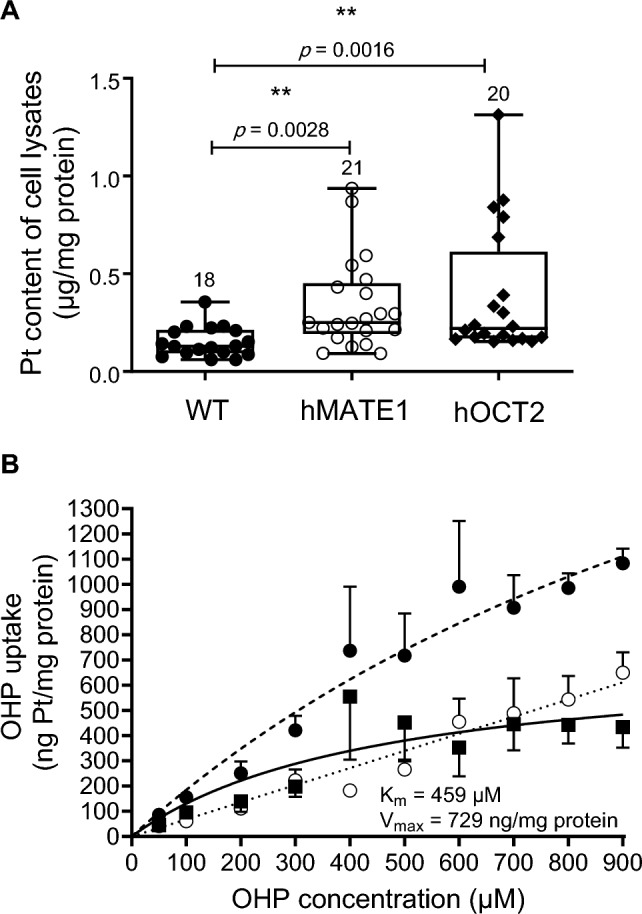
Panel A shows the intracellular accumulation of Platinum (Pt) in HEK293-WT, -hMATE1 and -hOCT2 cells measured by ICP-MS after 10 min incubation with 100 µM OHP. The numbers on the columns indicate the numbers of replicates measured in at least 6 independent experiments. The stars represent a statistically significant difference (*p* < 0.05) between the indicated groups (Kruskal–Wallis test with Dunn's multiple comparison post-test). The medians with their ranges and the exact *p* values are also shown. Panel B shows the specific OHP uptake by hOCT2-HEK293 cells (depicted as open circles) assessed by exposing the cells to increasing concentrations of OHP (50–900 µM) and measuring Platinum concentration in cell lysates after a 10-min incubation with OHP. The specific uptake was determined by subtracting the unspecific uptake (illustrated as closed squares), calculated in the presence of 1 mM TPA^+^, from the total uptake (depicted as closed circles). Each data point represents the mean ± SEM of four independent measurements (*N* = 4). The specific OHP-uptake rate exhibited saturation (solid line), enabling the determination of *K*_*m*_ and *V*_max_ values, which were determined to be 459 µM and 729 ng/mg protein, respectively

First, the ability of OHP to inhibit the uptake of the fluorescent organic cation ASP^+^ in HEK293 cells expressing hOCT1, hOCT2, hMATE1, mOCT1 or mOCT2 was evaluated (Fig. 2). Surprisingly, OHP in the concentration range 10^–6^ to 10^–3^ M showed no inhibition of ASP^+^ uptake. Rather, a slight stimulation of ASP^+^ uptake was observed at low OHP concentrations. Since these data did not show an interaction of OHP with any OCT paralog and ortholog, contrary to the results in the literature (Zhang et al. 2006), the accumulation of OHP in HEK293 cells expressing hOCT2 or hMATE1 was evaluated in comparison to that measured in HEK293 cells transfected with empty vector (referred to as wildtype (WT) cells) after 10 min incubation with 100 µM OHP using ICP-MS (Fig. 3A).

**Fig. 4:**
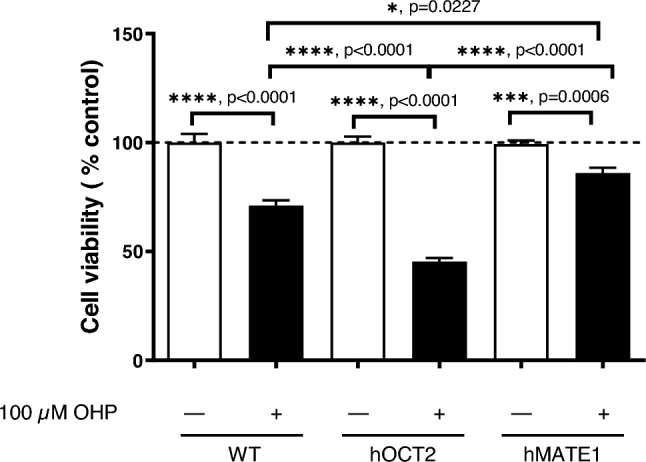
Comparison of cytotoxic effect of 10 min incubation with OHP (100 µM) followed by 48 h post-incubation time with OHP-free medium in WT-, hOCT2-, and hMATE1-HEK293 cells. Each bar shows the mean ± SEM of 12 replicates, measured in at least 3 independent experiments. In each cell line tested, treatment with OHP caused a significant reduction of cell viability (****p < 0.0001, and ***p = 0.0006, Mann Whitney test). The effect measured in hOCT2-HEK293 cells was significantly stronger than that measured in WT- and hMATE1-HEK293 cells (****p < 0.0001 Kruskal–Wallis test with Dunn's multiple comparison post-test). OHP toxicity in hMATE1-HEK293 cells was significantly milder that that measured in WT-HEK293 cells (*p = 0.0227 Kruskal–Wallis test with Dunn's multiple comparison post-test)

The results of these experiments confirm that both hMATE1 and hOCT2 mediate the cellular accumulation of OHP. Figure 3B depicts the outcomes of saturation experiments, revealing that OHP is indeed transported by hOCT2, with a K_m_ value of 459 µM and a V_max_ value of 729 ng/mg protein. To investigate the role of this transport in the cellular toxicity of OHP, WT-, hMATE1, and hOCT2-HEK293 cells were incubated with 100 µM OHP for 10 min. The incubation solution was then replaced with normal medium and cell viability was measured by MTT assay at 48 h post-incubation (Fig. 4). Control experiments performed without OHP were set to 100%. Incubation with 100 µM OHP decreased cell viability in each cell line tested. However, the expression of hOCT2 was associated with much greater cell damage than that observed in the other cell lines. Conversely, OHP caused less toxicity in hMATE1-HEK293 cells, probably because hMATE1 can mediate OHP efflux from the cells during the post-incubation period when the incubation solution was OHP-free. While cell acidification led to an increase in OHP toxicity measured in hMATE1-HEK293 cells, it is noteworthy that this maneuver also resulted in a decrease in the viability of control cells. This indicates that the acidification process itself had an impact on cell viability, and its effects should be carefully considered when interpreting experimental outcomes related to cellular responses and toxicity (see Supplementary Materials, Figure S4).

**Fig. 5:**
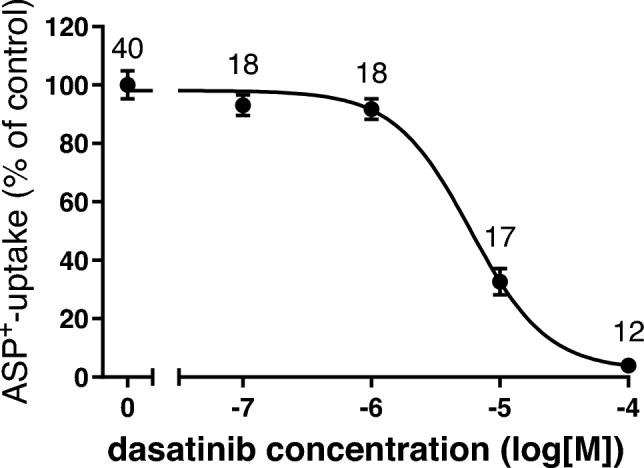
Effect of increasing concentrations of dasatinib on the uptake of 1 µM ASP^+^ in HEK293-hOCT2 cells, expressed as mean ± SEM compared to that measured in the absence of dasatinib (= 100%). The numbers above each measured concentration indicate the number of replicates measured in three independent experiments. The IC_50_ of dasatinib for ASP^+^ uptake by hOCT2 is 5.9 µM (logIC_50_ = −5.2 ± 0.1).

Since it has been proposed that the tyrosine kinase inhibitor (TKI) dasatinib may be able to compete with OHP for transport by hOCT2 and to protect non-target cells from unwanted OHP toxicity (Huang et al. 2020), the interaction of dasatinib with hOCT2 was functionally characterised. Dasatinib inhibited the ASP^+^ uptake by hOCT2 in a concentration-dependent manner with an IC_50_ of 5.9 µM (Fig. 5).

**Fig. 6:**
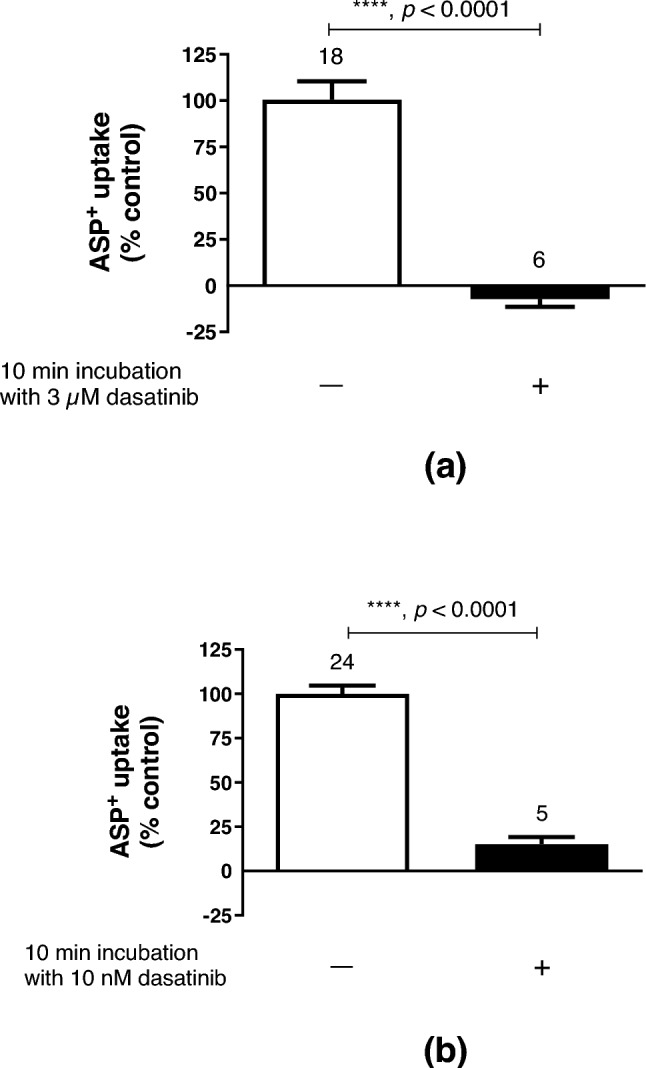
Effect of 10 min incubation with 3 µM (panel **a**) or 10 nM (panel **b**) dasatinib ( +) on ASP^+^ uptake in HEK293-hOCT2 cells. Control experiments performed after 10 min incubation with RLS, followed by measurement of ASP^+^ uptake, were set to 100%. 10 min incubation with 3 µM dasatinib completely suppressed ASP^+^ uptake by hOCT2 (−7.2 ± 4.3% of total uptake). 10 min incubation with 10 nM dasatinib also resulted in a strong decrease of ASP^+^ uptake by hOCT2 (to 15.2 ± 4.0% of total uptake). Each column represents the mean ± SEM. The numbers above the columns indicate the number of replicates measured in at least 3 independent experiments. Statistical significance was calculated using a Mann–Whitney test, asterisks together with the *p*-values indicate the significance level

**Fig. 7:**
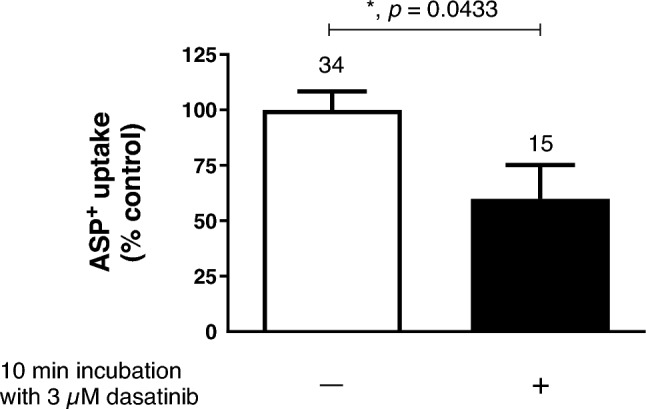
Effect of 10 min incubation with 3 µM dasatinib ( +) on ASP^+^ uptake in HEK293-hOCT2-Y544A cells. Control experiments performed after 10 min incubation with RLS, followed by measurement of ASP^+^ uptake, were set to 100%. 10 min incubation with 3 µM dasatinib significantly inhibited ASP^+^ uptake by hOCT2-Y544A (to 59.8 ± 15.4% of total uptake). Each column represents the mean ± SEM. The numbers above the columns indicate the number of replicates measured in at least 3 independent experiments. Statistical significance was calculated using a Mann–Whitney test, the asterisk together with the *p*-values indicate the significance level. This effect was significantly weaker (*p* = 0.03, Mann–Whitney test) than that observed under the same condition for the wild-type form of hOCT2 (reduction to −7.2 ± 4.3% of total uptake, see Fig. 6a)

It is known that OCT activity can be rapidly regulated by several intracellular signalling pathways and kinases (Ciarimboli and Schlatter 2005). Therefore, it was investigated whether short-term (10 min) incubation with the TKI dasatinib could alter ASP^+^ uptake by hOCT2. Both 10 min incubation with 3 µM (a concentration in the IC_50_ range of direct dasatinib inhibition of ASP^+^ uptake by hOCT2 as shown in Fig. 5) and with a lower (10 nM) concentration of dasatinib strongly inhibited ASP^+^ uptake by hOCT2 to -7.2 ± 4.3% and 15.2 ± 4.0% of control uptake, respectively (Fig. 6a, b), suggesting that dasatinib can regulate hOCT2 activity. By scanning the hOCT2 amino acid sequence with Prosite (Sigrist et al. 2012), a unique tyrosine kinase phosphorylation site was detected at position 544 (Y544). Therefore, Y544 in hOCT2 was replaced by alanine and transporter regulation by dasatinib was again investigated (Fig. 7). Incubation of Y544A mutants with 3 µM dasatinib for 10 min resulted in an inhibition of ASP^+^ uptake to 59.8 ± 4.0% of the control uptake. This inhibition of ASP^+^ uptake was significantly lower than that measured in wild-type hOCT2 (*p* = 0.03, Mann–Whitney test), suggesting an important role for Y544 phosphorylation in hOCT2 regulation.

**Fig. 8:**
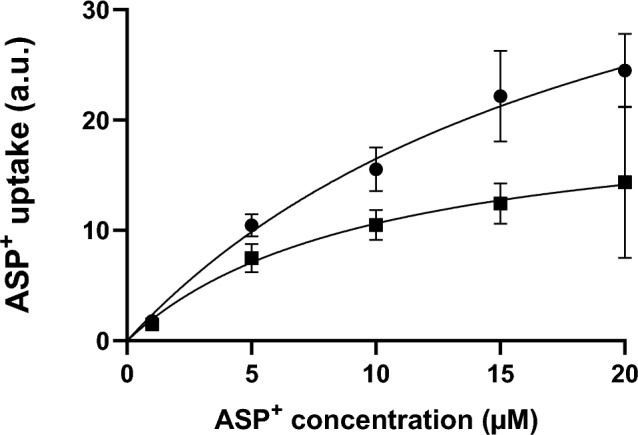
Effect of 10 min incubation with 10 nM dasatinib (closed squares) on saturation of ASP^+^ uptake in HEK293-hOCT2 cells. Control experiments (closed dots) were performed after 10 min incubation with RLS, followed by measurement of ASP^+^ uptake. 10 min incubation with 10 nM dasatinib significantly changed the *V*_max_ of ASP^+^ uptake (from 50.3 ± 9.1 fluorescence arbitrary units, a.u., with 28 degrees of freedom (DF) to 21.2 ± 4.7 a.u. with 22 DF, after control and dasatinib incubation, respectively, *p* = 0.0126, unpaired t-test). The affinity changes of hOCT2 after dasatinib incubation were not statistically significant different (from 20.5 ± 6.3 µM with 28 DF to 9.9 ± 5.1 µM with 22 DF, after control and dasatinib incubation, respectively). Each experimental point represents the mean ± SEM. Every ASP^+^ concentration was measured at least in 6 replicates

In an attempt to determine the regulatory mechanism of dasatinib on hOCT2-mediated ASP^+^ transport, saturation curves of ASP^+^ uptake were measured after 10 min incubation with RLS (control experiments) or 10 nM dasatinib (Fig. 8). The results of these experiments show that incubation with dasatinib significantly decreased the *V*_max_ of hOCT2, suggesting an effect of dasatinib on cellular trafficking of the transporter to/from the plasma membrane.

**Fig. 9:**
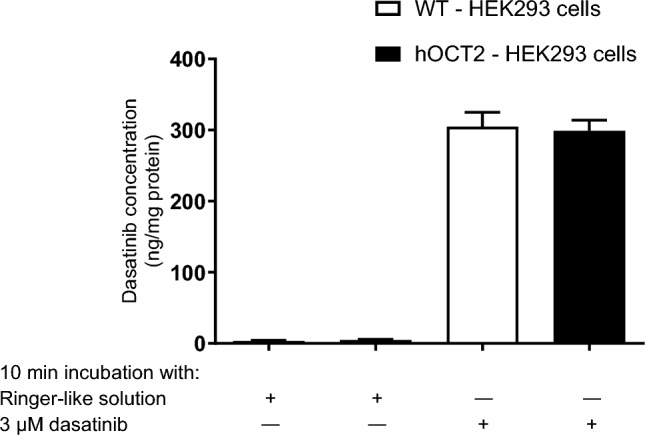
Dasatinib accumulation measured by LC–MS in cell lysates from WT- and hOCT2-HEK293 cells after 10 min incubation with RLS or 3 µM dasatinib. Data are expressed as mean ± SEM of 6 independent experiments. No difference in dasatinib accumulation was observed between WT—and hOCT2—HEK293 cells, indicating that dasatinib is not a substrate of hOCT2

To investigate whether dasatinib is a substrate of hOCT2, we compared its cellular accumulation in WT- and hOCT2-HEK293 cells after 10 min incubation with 3 µM dasatinib. Cellular dasatinib levels were measured by LC–MS. As no preferential accumulation of dasatinib was observed in HEK293 cells stably expressing hOCT2 (Fig. 9), it was concluded that dasatinib is a non-transported inhibitor of hOCT2.

**Fig. 10:**
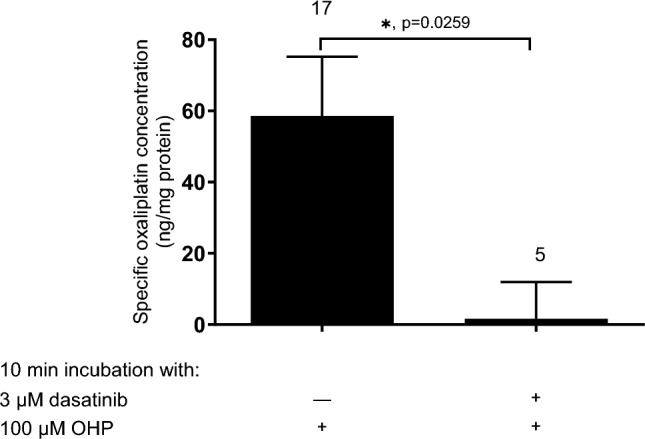
Transporter-specific OHP accumulation measured by ICP-MS in cell lysates from HEK293-hOCT2 cells after 10 min incubation with 100 µM OHP alone or together with 3 µM dasatinib. To calculate the hOCT2-specific OHP accumulation, the cellular OHP content measured under the same experimental conditions in WT HEK293 cells (144.2 ± 15.4 ng OHP/mg protein, *n* = 24 and 132.0 ± 15.0 ng OHP/mg protein, *n* = 5 without and with dasatinib, respectively) was subtracted from the content measured in hOCT2-HEK293 cells. Data are expressed as mean ± SEM. The numbers above the columns indicate the number of independent experiments. Addition of dasatinib significantly reduced hOCT2-specific OHP accumulation (*p = 0.0405, Mann Whitney test)

In further experiments, the effect of co-administration of dasatinib on OHP cellular accumulation was investigated using ICP-MS (Fig. 10). OHP cellular accumulation was significantly reduced by co-incubation with dasatinib.

**Fig. 11:**
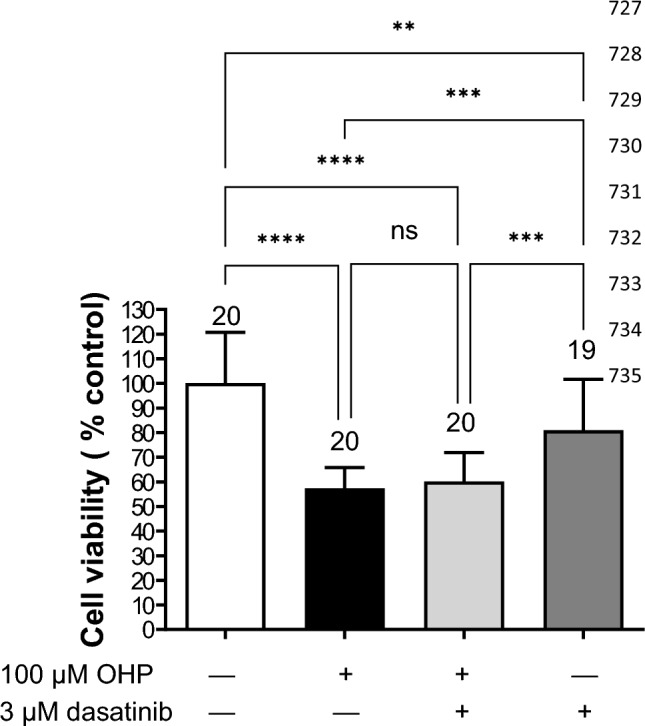
Cytotoxic effect of 10 min incubation with OHP (100 µM) in the presence or not of 3 µM dasatinib followed by 48 h post-incubation with OHP- and dasatinib-free medium in hOCT2 cells. Each bar shows the mean ± SEM of 20 and 19 replicates from 3 independent experiments. Statistical analysis was performed using an ANOVA-test with Dunnett’s multiple comparison test. ****, ***, and ** indicate a statistically significant difference with *p* < 0.0001, *p* = 0.007, and *p* = 0.022, respectively. Co-incubation with dasatinib was not able to reduce OHP-cellular toxicity. Incubation with dasatinib alone caused also a significant cellular toxicity (**p = 0.022)

The potential protective role of dasatinib against OHP-induced cellular toxicity was investigated by measuring cell viability assessed by the MTT assay in cells incubated with 100 µM OHP alone or together with 3 µM dasatinib for 10 min, followed by a post-incubation period of 48 h in OHP- and dasatinib-free medium (Fig. 11). Co-incubation with dasatinib did not reduce OHP cell toxicity. Incubation with dasatinib alone induced a significant decrease in cell viability.

## Discussion

Oxaliplatin (OHP) is well known for its effectiveness in treating colorectal cancer and is a crucial component of combination chemotherapy used at various stages of the disease, including both adjuvant and metastatic settings. However, the usage of OHP has been associated with the development of severe peripheral neurotoxicity (OHP-induced peripheral neurotoxicity (OIPN)), which negatively impacts the quality of life for cancer survivors. Thus, understanding the mechanisms responsible for inducing OIPN is essential to establish protective therapy protocols. In this study, our focus was on characterizing the in vitro interaction between OHP and the human organic cation transporter 2 (hOCT2), given its role in facilitating OHP uptake into cells (Zhang et al. 2006). Additionally, we investigated OHP interaction with the human multidrug and toxin extrusion protein 1 (hMATE1). The hMATE1 also plays a role in mediating the movement of OHP across the plasma membrane, for example influencing renal OHP tubular secretion into the urine (Yonezawa et al. 2006). The efficient secretion of OHP into urine by the renal MATE transporters, as opposed to the less effective secretion of the platinum derivative cisplatin, contributes significantly to the discrepancy in nephrotoxicity between the two compounds. Due to its superior elimination through the MATE transport system, OHP exhibits lower nephrotoxicity compared to cisplatin (Yokoo et al. 2007). Through a mass spectrometry analysis comparing OHP accumulation in cells overexpressing hOCT2 or hMATE1, we demonstrated a clear involvement of both transporters in facilitating OHP transport into the cells, confirming that OHP is a substrate for these transporters. Interestingly, OHP did not inhibit the uptake of the fluorescent organic cation 4-(4-diethylaminostyryl)-N-methylpyridinium (ASP^+^) by OCT or hMATE1 into the cell. ASP^+^ is a well-known substrate for transporters of organic cations (Mehrens et al. 2000; Biermann et al. 2006; Minematsu and Giacomini 2011). Therefore, these results suggest that hOCT2 and hMATE1 may simultaneously transport ASP^+^ and OHP. Evidence for the simultaneous transport of two different substrates by OCT has already been provided in Koepsell (2019), suggesting the ability of certain organic cation transporters to perform co-transport in the same direction. The binding pocket of OCT has already been described as a large structure, with different, partially overlapping binding domains for different substrates (Busch et al. 1996; Ciarimboli et al. 2005; Koepsell 2019; Keller et al. 2019). This feature of the binding pocket may explain the polyspecificity of OCT. Saturation experiments showed that OHP is transported by hOCT2 with a *K*_*m*_ of 459 µM. Such extremely OHP concentrations may not be attained in the blood of patients undergoing OHP treatment. Nevertheless, depending on the specific dosing protocols and administration routes, certain tissues may experience even higher OHP concentrations. For instance, in cases where peritoneal administration of 460 mg/m^2^ OHP is employed, a peak plasma concentration of 30 µM OHP has been measured. Notably, the peritoneal concentration of OHP was found to be 25 times higher than that in the plasma (Elias et al. 2002).

Dasatinib interacts with transporters for organic cations (Minematsu and Giacomini 2011; Sprowl et al. 2016) and has been proposed as a protecting substance against OIPN (Huang et al. 2020). Indeed, there is a clinical trial in the recruiting phase intended to evaluate the efficiency of dasatinib for “Prevention of Oxaliplatin-Induced Neuropathy in Patients With Metastatic Gastrointestinal Cancer Receiving FOLFOX” (https://classic.clinicaltrials.gov/ct2/show/NCT04164069, accessed 2023-7-27). Therefore, we have investigated the interaction of dasatinib with the ASP^+^ transport mediated by hOCT2, confirming a high-affinity inhibition of ASP^+^ uptake by dasatinib with an IC_50_ of 5.9 µM, very closed to the 2 µM value previously reported in (Minematsu and Giacomini 2011). However, dasatinib interaction with hOCT2 is not only limited to a competition with ASP^+^ uptake: dasatinib can also rapidly regulate hOCT2 activity: 10 min incubation with 3 µM dasatinib (a concentration in the IC_50_ range for direct inhibition of ASP^+^ uptake) completely suppressed hOCT2 transport. Decreasing dasatinib concentration to 10 nM still produced more than 80% inhibition of transporter activity. It has been already demonstrated that tyrosine phosphorylation is a regulation mechanism of OCT activity (Biermann et al. 2006; Ciarimboli et al. 2005; Sprowl et al. 2016). By comparing the presence of tyrosine phosphorylation sites of the type [RK]-x(2)-[DE]-x(3)-Y in human hOCT1 (O15245), hOCT2 (O15244) and hOCT3 (O75751) using ProSite (Sigrist et al. 2012), a potential phosphorylation site was found only in hOCT1 and hOCT2 at position 543 (Y543) and 544 (Y544), respectively, but not in the hOCT3 amino acids sequence. Regulation of the hOCT2 variant Y544A by dasatinib was much weaker than that observed for WT-hOCT2, suggesting that dasatinib inhibits hOCT2 phosphorylation at position Y544. Therefore, among other previously identified Y sites (Sprowl et al. 2016), Y544 also appears to be involved in transporter regulation by tyrosine kinases. Indeed, dasatinib was not able to regulate the activity of hOCT3 (supplementary Materials Fig. 3). Since dasatinib incubation significantly reduced the V_max_ of hOCT2, tyrosine phosphorylation at position Y544 may change hOCT2 trafficking to/from the plasma membrane.

To investigate whether dasatinib functions as a substrate of hOCT2, we conducted a comparison of its accumulation in both WT- and hOCT2-HEK293 cell lines. Surprisingly, we observed no significant difference in dasatinib's intracellular concentration between these two cell lines. This finding strongly indicates that dasatinib does not rely on hOCT2 for transport and instead acts as a non-transported inhibitor and negative regulator of the hOCT2. Indeed, we found that dasatinib had a significant impact on the cellular accumulation of OHP mediated by hOCT2. Specifically, dasatinib was observed to substantially reduce the intracellular accumulation of OHP by hOCT2. These results indicate that dasatinib acts as a non-transported inhibitor of hOCT2-mediated transport, leading to decreased cellular accumulation of OHP.

Cell viability measurements after 10 min incubation with 100 µM OHP or with 3 µM dasatinib, or a combination of both, did not show protection against OHP cellular toxicity by dasatinib, probably because dasatinib itself is toxic to cells. It should be noted that in this setup, HEK293 cells stably expressing hOCT2 are proliferating cells and therefore may be sensitive to inhibition of proliferation by dasatinib, even though cell toxicity experiments were performed at 80–90% confluency. Indeed, the use of cimetidine, which is an hOCT2 inhibitor without affecting cell proliferation, provided protection against OHP cell toxicity in vitro (see Supplementary Fig. 2). Conversely, in vivo DRG neurons damaged by OHP treatment are differentiated cells and here the inhibitory effects of dasatinib on OHP cellular uptake may prevail.

In conclusion, this work demonstrated that OHP is a substrate of hOCT2 and hMATE1. The cellular uptake mediated by hOCT2 is critical for the development of OHP cellular toxicity, while transport by hMATE1 may have a protective role. The tyrosine kinase inhibitor dasatinib is a non-transported inhibitor and regulator of hOCT2 activity, which can reduce the cellular accumulation of OHP. Whether dasatinib can protect non-proliferating cells from OHP toxicity should be investigated in a more appropriate model.

### Supplementary Information

Below is the link to the electronic supplementary material.

Supplementary file1 (DOCX 210 KB)

Supplementary file2 (VCF 0 KB)
