# Supplementary material for: Interaction of the chemotherapeutic agent oxaliplatin and the tyrosine kinase inhibitor dasatinib with the organic cation transporter 2
Source: Arch Toxicol. 2024 Apr 8;98(7):2131–42. doi: 10.1007/s00204-024-03742-1 (PMC11169033; doi:10.1007/s00204-024-03742-1)
Supplement: Supplementary file 1 — Supplementary file1 (DOCX 210 KB) [file 204_2024_3742_MOESM1_ESM.docx]

Supplementary Materials of manuscript:

Interaction of the chemotherapeutic agent oxaliplatin and the tyrosine kinase inhibitor dasatinib with organic cation transporter 2: a potential way to reduce undesired oxaliplatin toxicity?

Sara Ahmed Eltayeb^1^, Julia M. Dressler^2^, Lukas Schlatt^2^, Moritz Pernecker^1^, Ute Neugebauer^1^, Uwe Karst^2^, Giuliano Ciarimboli^1^

^1^Medizinische Klinik D, Experimentelle Nephrologie, Universitätsklinikum Münster, Münster, Germany; ^2^Institut für Anorganische und Analytische Chemie, Westfälische Wilhelms-Universität, Münster, Germany

Table S1. Primer for Y544A substitution:

Forw., 5’-3’: GAA AAG ATG ATT GCC CTC CAA GTT CAG AAA CTA G

Rev., 5’-3’: CTA GTT TCT GAA CTT GGA GGG CAA TCA TCT TTT C

Figure S1: Comparison of cytotoxic effect of 10 minutes incubation with OHP (10^-6^ to 10^-3^ M) followed by 48 h post-incubation time with OHP-free medium in hOCT2-HEK293 cells. Each point shows the mean ± SEM of 12 replicates, measured in at least 3 independent experiments. Treatment with 10^-5^-10^-3^ M OHP caused a significant reduction of cell viability (*** *p* = 0.0002, and **** *p* < 0.0001, ANOVA with Tukey’s multiple comparison test) with 50% of the effect (EC_50_) at a 50 µM OHP concentration.

Figure S2: Comparison of cytotoxic effect of 10 minutes incubation with RLS, 100 µM cimetidine, 100 µM OHP, and 100 µM cimetidine + 100 µM OHP followed by 48 h post-incubation time with OHP-free medium in hOCT2-HEK293 cells. Each column shows the mean ± SEM of 8 replicates, measured in 2 independent experiments. Treatment with 100 mM M OHP caused a significant reduction of cell viability (** *p* = 0.0045, ANOVA with Dunnett’s multiple comparison test), which could be prevented by co-incubation with 100 µM cimetidine. Incubation with 100 µM cimetidine alone did not change cell viability.

Figure S3: Effect of 10 min incubation with 0.1 or 1 µM dasatinib on ASP^+^ uptake in HEK293-hOCT3 cells. Control experiments performed after 10 min incubation with RLS, followed by measurement of ASP^+^ uptake, were set to 100%. 10 min incubation with dasatinib did not change ASP^+^ uptake by hOCT3. Each column represents the mean ± SEM. The numbers above the columns indicate the number of replicates measured in at least 3 independent experiments.

Figure S4: Effect of intracellular acidification on OHP toxicity in HEK293-hMATE1 cells. Each column is presented as mean ± SEM of three independent experiments. After incubation with 30 mM NH_4_Cl for 30 minutes, incubation solution was removed and replaced with fresh RLS containing 100 µM OHP or not (control experiments). After 5 minutes, OHP or control RLS were replaced by normal medium, and cells were incubated for 48 h before measuring cell viability by an MTT assay. Cell viability in control cells without acidification was set to 100%. Incubation with OHP significantly decreased cell viability both with and without acidification (**** p = 0.0001, Anova with Tukey's multiple comparison test). Acidification significantly (**** p = 0.0001, Anova with Tukey's multiple comparison test) increased OHP toxicity. However, acidification itself caused a significant decrease of cell viability (**** p = 0.0001, Anova with Tukey's multiple comparison test).
